# Seroepidemiological study of Q fever in Lorestan province, western Iran, 2014

**Published:** 2017-08

**Authors:** Mohammad Hassan Kayedi, Hamid Mokhayeri, Mehdi Birjandi, Ali Chegeni-Sharafi, Saber Esmaeili, Ehsan Mostafavi

**Affiliations:** 1 Department of Parasitology, School of Medicine, Lorestan University of Medical Sciences, Khorramabad, Iran; 2 Department of Communicable Diseases Control and Prevention, Health Center, Lorestan University of Medical Sciences, Khorramabad, Iran; 3 Department of Epidemiology, School of Public Health, Lorestan University of Medical Sciences, Khorramabad, Iran; 4 Department of Epidemiology and Biostatistics, Research Centre for Emerging and Reemerging Infectious Diseases, Pasteur Institute of Iran, Tehran, Iran; 5 National Reference Laboratory for Plague, Tularemia and Q Fever, Research Centre for Emerging and Reemerging Infectious Diseases, Pasteur Institute of Iran, Akanlu, Kabudar Ahang, Hamadan, Iran; 6 Department of Bacteriology, Faculty of Medical Sciences, Tarbiat Modares University, Tehran, Iran

**Keywords:** *Coxiella burnetii*, Livestock, Seroprevalence, ELISA, Western Iran

## Abstract

**Background and Objectives:**

Q fever is a zoonotic disease and farm animals serve as the main reservoir of the disease. The aim of this study was to evaluate the seroprevalence of Q fever in sheep, in Lorestan province in western Iran.

**Materials and Methods:**

In this cross-sectional study, 330 blood samples were collected from sheep, from each county in Lorestan province. The samples were tested by ELISA for the presence of immunoglobulin (IgG) against *Coxiella burnetii*.

**Results:**

Among the samples tested, 45 samples (13.64%) were seropositive. Of 35 studied herds, 21 (60%) had a history of infection. In terms of number of positive samples, there was no significant difference between the three geographical regions (central, west and east) (p=0.687). There was no statistically significant difference between age groups (p =0.604). Gender also had no effect on infection rates, in female and male sheep (p =0.814). No significant difference was observed between the number of lactation and positive serology (p =0.376). The rate of infection with Q fever and abortion also had no statistically significant difference (p =0.152).

**Conclusion:**

The findings of this study showed that sheep in Lorestan were infected by Q fever and the cycle of disease transmission had been established between animals and ticks.

## INTRODUCTION

Q fever is a zoonotic disease caused by a bacterium *C. burnetii* (1). The bacterium has been separated from a variety of domestic and wild animals, birds, mammals and arthropods. Sheep, goats and cattle are considered the main reservoir of the disease. Also, 40 species of ticks (Ixodidae and Argasidae families) can transmit the disease to animals (2, 3). Infected animals discharge the bacteria through fetal fluids during birth delivery and also through breast milk, urine, feces and reproductive discharges. Q fever is transferred among cattle through tick bites, inhalation or ingestion of infected droplets and direct or indirect contact with infected animals or their discharges. *C. burnetii* infection in ruminants occurs at any age and usually has no obvious symptoms, but it can cause miscarriage in sheep and goats. The disease in cattle also causes miscarriage, stillbirth, reduced-fertility, dystocia and mastitis (1, 4). High-risk persons, such as veterinarians, farmers, farm workers and slaughterhouse workers may be infected through inhalation or ingestion of infected droplets, direct or indirect contact with discharges from infected animals or the consumption of raw milk, but they rarely become infected through tick bites (1, 5).

In Iran, the first clinical case of acute Q fever in humans was reported in 1952 (6). After 1976, the disease was forgotten in Iran and no human cases were reported (7). Phase one and two antibodies against *C. burnetii* were once again observed in suspected patients with fever in 2009, in Kerman (southeastern Iran) (5). This research marks the beginning of the seroepidemiological studies of Q fever in different animals and human populations (8–16).

One of the oldest reports of infection with Q fever in Iran was published in 1976 in Lorestan province, western Iran and positive cases of infection with *C. burnetii* in cattle, sheep and goats had been reported as 7%, 3.2% and 1.7%, respectively. No further research has been carried out on the Q fever cases in the province (14). However, due to the common nature of this disease among humans and animals, as well as the role of this disease in abortion in livestock, it is necessary to determine the seroprevalence of the disease in animals, in this province.

## MATERIALS AND METHODS

### Studied area.

The present study was conducted in 2014, in Lorestan province in western Iran. Lorestan covers an area of 28,175 square kilometers, which constitutes 1.73% of the country. The northern neighbors are the provinces of Hamadan and Arak, southern are Khuzestan and Chaharmahal-Bakhtiari, western are Kermanshah and Ilam and eastern is Isfahan. The province has 10 counties and approximately 2 million people, of which about 1.2 million live in urban areas and about 800,000 in the rural areas. The counties of Aligodarz, Azna and Doroud are in the east of province, the counties of Khorramabad, Boroujerd and Alashtar are in the central region and the counties of Chegeni, Delfan, Kohdasht and Poledokhtar are located in the western region. The province has 6.5 million farm animals (5.5% of the national livestock population) and the province is ranked sixth among all the provinces in Iran. Seventy two percent (72%) of the livestock are small herds (sheep and goats).

### Samples collection.

**Fig. 1.**
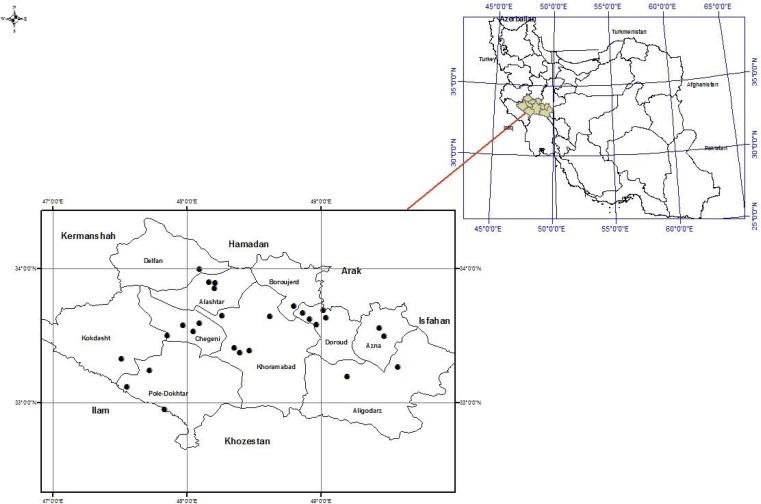
Map of Lorestan province, sampling regions of counties and samples from sheep are marked on the map.

In this cross-sectional study, the veterinary organization at Lorestan province collaborated in blood samples collection from sheep in the province. At least two areas in the outskirts of the cities and 2 to 4 rural areas were selected in each of the 10 counties in the province (Fig. 1). In the selected areas, sheep were randomly selected and recruited. From 10 counties, 35 villages were randomly selected and sheep blood samples were collected from one herd in each village.

After obtaining consent from the owner of the livestock and documenting the necessary information such as gender, age, name of village, the number of pregnant animals and the number of abortions, 5 ml of venous blood was carefully collected, using a venoject blood collection system, using the neck jugular vein of the animals. The samples were then placed in a cold box and sent to the central laboratory of the provincial Veterinary office, shortly after blood collection, under cold chain conditions. In the laboratory, the samples were centrifuged for 10 minutes at 3,000 rpm and sera from the samples were isolated and frozen at −20°C. Serum samples were sent to the Research Center for Emerging and Reemerging Infectious Diseases at the Pasteur Institute of Iran (National reference laboratory for Q fever, plague and tularemia), while maintaining the cold chain for serologic studies.

### Serology.

The serum samples were tested for the detection of antibodies against *C. burnetii* (IgG) using a commercial ELISA kit (Checkit Q fever IDEXX Laboratories), in accordance with the instructions provided by the manufacturer. Based on the obtained optical density (OD) three groups has been formed: positive, border-line and negative.

### Statistical analysis.

Statistical analysis was performed using SPSS version 20 and the statistical significance level of p <0.05 was considered significant. The correlation between risk factors and seroprevalence was evaluated, using chi-square. Risk factors were as follows: age, gender, number of parturition, abortions, location, and whether the animal was native or imported.

## RESULTS

Among the 330 sheep’s included in the study, 286 animals (86.67%) were female and 311 animals (94.24%) were native. The age of most of the studied sheep was 3 to 4 years (181, 12.70%). In terms of the number of birth delivery, the majority of sheep (151 animals) had delivered 2 or 3 times (9.93%). Most of them (186 animals) had no abortion.

Additionally, 45 sheep (13.64%) tested positive for IgG for Q fever and 23 animals (6.97%) were border-line. Twenty-one of the 35 herds (60%) were seropositive (Table 1). There was no significant difference between the three geographical regions (central, western and eastern) (p =0.687). There was no significant difference between the three counties of Aligudarz, Azna and Doroud in the eastern region of the province (p =0.012), while the lowest level of seropositive cases was observed in Aligudarz county (6.98%). In terms of geographical distribution, the highest and lowest levels of infection were in the samples belonging to the counties of Doroud (32.26%) and Kohdasht (6.90%), respectively (Table 1).

**Table 1.** Percentage of positive cases of Q fever in sheep in different geographical areas of Lorestan province, Iran.

**Area**	**Number of sheep**	**Percentage of seropositive**	**Name of county**	**No. of villages from which samples were taken**	**Number of blood samples (positive)**	**Percentage of seropositive %**	**p- Value**
East	92	16.30	Aligudarz	6	43 (3)	6.98	0.012
			Azna	2	18 (2)	11.11	
			Doroud	3	31 (10)	32.26	

Center	122	12.30	Boroujerd	3	28 (5)	17.86	0.482
			Khorramabad	7	68 (6)	8.82	
			Alashtar	4	26 (4)	15.38	

West	116	12.93	Chegeni	2	27 (6)	22.22	0.080
			Delfan	2	28 (2)	7.14	
			Kohdasht	1	29 (2)	6.90	
			Poledokhtar	5	32 (3)	15.63	

Total	330	13.64		35	330 (46)	13.64	

The age group with the highest infection rate was 5 to 6-year olds (15.85%) and the lowest was in the age group of 7+ years (12.5%). There was no significant difference between age groups (p =0.604) (Table 2). Gender also had no effect on infection rates in female (13.99%) and male (11.36%) sheep (p =0.814). No significant difference between the number of birth delivery and positive serology has been observed, among 286 female sheep (p =0.376). Among 311 sheep native to Lorestan province, 13.5% were seropositive and among 19 imported animals (from other provinces), 15.8% were seropositive (p =0.726). There was no significant difference between disease and abortion (p =0.152) (Table 2).

**Table 2.** Percentage of positive cases of Q fever in sheep in Lorestan province, Iran, in terms of age, number of birth delivery and abortions

**Studied feature**	**Studied classes**	**No. of blood samples**	**Percentage of seropositive %**	**p- Value**
Age	Lower or equal to 2 years	70	14.28	0.604
	3 to 4 years	181	12.70	
	5 to 6 years	63	15.85	
	7 years and older	16	12.50	

Number of parturitions	1 or less	40	15.00	0.376
	2 to 3	151	9.93	
	4 to 5	82	18.29	
	More than 6	13	7.69	

History of miscarriage	With no history	186	65.03	0.152
	Once	80	27.97	
	Twice	15	5.25	
	Three times and more	5	1.75	

## DISCUSSION

Our data show that the serological prevalence of Q fever in the sheep recruited in this project was 13.64%. A comparison between the results of our study and the data of the research study from 1976 on sheep in the province (3.2% infection), indicates a significant increase in the incidence of the disease among sheep in the province, during the period of 39 years (14).

In the present study, the seroprevalence of Q fever in sheep (13.64%) was lower compared to other studies, included 19.5% in central, northeastern and southwestern Iran (10), 33.6% in northwestern Iran (Ardebil province) (7), 23.7% in northern Iran (Mazandaran province) (8), 29.42% in southeastern Iran (11), and 33.9% in southeastern Iran (12) The seroprevalence was also higher compared to the study by Pour Mehdi Borougeni et al. (2014) with seroprevalence of 13.18% in the county of Ahvaz (13). In most regions of Iran, the prevalence of Q fever was higher than the present study; which may arises as a consequence of importing infected livestock from our eastern neighbors and transferring them to the eastern, central and northern provinces of country. Additionally, as ticks play a major role in the transmission of the disease from infected to healthy animals, a possible reason for the high prevalence in other provinces may be due to higher tick infection between livestock in other provinces in comparison with livestock in Lorestan, as no imported livestock found in Lorestan.

In a study conducted in 2012 and 2013 in India, a low seropositive prevalence in sheep (1.85%) has been reported (17). In 2013, a low seropositive prevalence in sheep in Sweden was also reported (0.6%) (18). In a study in Turkey (neighboring northwestern Iran), carried out in 2000, a 10.5% prevalence of the disease in sheep was reported. The results of these studies show that the prevalence of the disease in sheep in Iran is relatively higher compared with other countries (19).

Although a higher infection rate was found in the east of the province, the infection rate in Aligudarz county was lower than other eastern counties of province (6.98%). This is due to the fact that Aligudarz county is mountainous with bad road condition which it is not suitable for importing livestock from other regions of the province or from other provinces.

In terms of age, the highest prevalence was observed in the age group of 5–6 years old (15.85%) and that is not consistent with the study in Ardabil province in which most seropositive sheep were between the ages of 3 and 4 years old (7). In the present study, no significant difference was observed between the age groups in terms of the history of Q fever infection. In another study, in northern Iran (Mazandaran province, 2010–2011) the age of the sheep did not affect the percentage of infection (8).

In the present study, gender had no effect on infection rates in female (13.99%) or male (11.36%) sheep. These results are consistent with those from northwestern Iran (Ardebil, 2011–2012) (7) and northern Iran (Mazandaran Province, 2010–2011) (8), stating that gender had no effect on the infection rates.

Poor roads condition made access to some villages in the province impossible. Future studies needed to be carried out in the province using other livestock (cattle and goats) and vectors of the disease (ticks). It is also suggested that future studies examine positive seroprevalence in humans regarding the zoonotic nature of the disease.

In conclusion, our data show that Q fever has been spread in sheep in Lorestan. A comparison of our data with the data from research study in 1976 showed that the positive cases of the disease have increased from 3.2% to 13.64%, over 39 years in Lorestan. Considering the economic losses of farmers (miscarriages, etc.) and the risk of disease transmission from animals to humans, control and prevention of this disease by the relevant organizations is necessary.
